# The Colorado Desert

**Published:** 1882-06

**Authors:** 


### THE COLORADO DESERT.

There is an old adage which says that Arizona was the last spot on earth to be created ; that Yuma is the outpost of the nether regions, and the hottest place in the world. Every one knows the old story of the two soldiers who, while stationed at Fort Yuma, died, and, going straight to Hades, returned in a short time for their blankets ! Be that as it may, there can be no doubt that parts of Southern California and Arizona are among the hottest regions in the world. Neither the Desert of Gobi in Asia nor the Great Sahara in Africa can be worse, in this respect, than their small relative, the Colorado Desert, in California. A protracted journey of some four weeks over this desert gave me an excellent chance to see it and its worst aspect, and I purpose trying to give others an account of its most interesting features.

The desert occupies almost the whole of the large county of San Diego. It is some one hundred and fifty miles long and fifty miles wide, and the Southern Pacific Railroad runs through the centre of it. About sixty miles from Los Angeles, the railroad encounters a very heavy grade, one hundred to one hundred and ten feet to the mile, and it continues for twenty-two miles. At the summit, known as San Gorgonio Pass, begins the descent into the desert, and every mile brings you to a more desolate country. At Whitewater Station, twenty miles from the summit, the desert commences in earnest. First a few flowers enliven the scene.

Large *Œnotheras,* three or four inches in diameter grow on small stalks five or six inches high. Large plants of *Abronia maritima,* with clusters of brilliant purple flowers, spread over the ground. A little *Gilia* (*G. Lemmoni*), with white corolla and yellow centre, adds its beauty to the scene; and the only shrub, *Larrea Mexicana,* or creosote plant, with yellow flowers and sticky leaves and branches, reminds you of the forests you have left behind.—*Popular Science Monthly.*

